# Eye, Ear, Nose, and Throat

**Published:** 1895-03

**Authors:** 


### *SELECTIONS AND ABSTRACTS.*

---

### EYE, EAR, NOSE, AND THROAT.

Edited by Dunbar Roy, A.B., M.D.

### Penetrating Wounds of the Eye.

In a very excellent article entitled, "Penetrating Wounds of the Eye, Due to Pieces of Copper, and Their Treatment," coming from the pen of Professor H. Leber, of Heidelberg, read before the International Ophthalmological Congress in Edinburgh and published in the *Revue Gen d' Ophth.*, some very practical points are clinically deduced as pertaining to the subject in question. Professor Leber is perhaps the oldest, besides being the weightiest, leading exponent of ocular pathology of the present day, and his leadership to this position is recognized throughout Germany. Although Heidelberg is comparatively a small town, yet on account of its being the home of this distinguished ophthalmologist and his most complete laboratory, more students and visiting ophthalmologists visit this town, especially those interested in ocular pathology, than any other city of continental Europe. The town itself is far-famed for its quaint beauty and delightful situation upon the rugged cliffs of the Rhine, and since its climate in summer is so fresh and invigorating, many students from all over Europe spend the heated months in doing laboratory work in this charming town. So that whenever a physician passes through Heidelberg in the summer, he should certainly stop and visit the medical laboratories, for he is almost sure to find a host of American students who are delighted to show him the place. But this is a digression, since my object is to condense the views of Professor Leber, which are held upon the subject above referred to. He believes that penetrating wounds of the eye due to copper are of a special kind. This metal can cause purulent inflammations by its chemical action alone, so that without the help of microbes, hypopyon and purulent infiltrations of the vitreous may result. Moreover, it is especially noxious to the retina and produces detachment or atrophic degeneration. In consequence of this dangerous character of these foreign bodies, it must be our principal aim in the treatment to extract them promptly. When the piece of copper lies in the anterior chamber, its extraction is not very difficult, but when in the posterior portion of the globe, the operation is much more difficult. However, with few exceptions, Professor Leber always attempts their removal, claiming that better results follow than the sacrificing of an eye immediately with enucleation.

"Out of 46 cases of lesions, due to the penetration of pieces of copper or brass into the eye, observed during twenty-three years, 6 concerned the anterior chamber and iris, 40 the posterior part of the eyeball, but 38 only were located. In 9 of these 38 I at once proceeded to enucleation, or exenteration. The removal of the foreign body was attempted in 25 cases, and in the remaining 4, I only made a preparatory iridectomy, or a cataract operation.

"Of the 25 cases in which the extraction was attempted, 18 were successful (72 per cent.), 7 were unsuccessful (28 per cent.); to this number we must add 6 successful extractions from the anterior chamber, so that in 24 out of 31 cases the foreign body was removed. In 2 of the 18 cases in which the foreign body was removed from tne posterior part of the eyeball, I have enucleated finally, and in a third case I have advised enucleation."

After further detailing the character of the inflammation, the author closes with this practical advice :

"I am convinced that it is wrong to enucleate an eye which incloses a piece of copper as long as the function of retina is intact; even in those cases in which vision cannot be restored, I believe that it is preferable to attempt to save the eye, since the danger of sympathetic ophthalmia is an exceptional one."

---

### LOW DEGREE LENSES.

A very interesting paper entitled, "Are Low Degree Lenses of Merely Mythical Value?" was read at the last meeting of the American Medical Association in the Section on Ophthalmology, by Dr. H. M. Starkey, of Chicago, which is of sufficient interest to warrant the tabulation in this department of the conclusions drawn by the author.

The paper in question was incited by the appearance of an editorial in *The Journal* strongly condemning the use of weak spheres and cylinders of low degrees, declaring "that the advocates of their use had failed to furnish evidence that their patients had been benefited by them." Dr. Starkey thereby attempted to refute this statement by addressing the following letter to one hundred ophthalmologists :

"DEAR DOCTOR :—There appears to be much difference of opinion among ophthalmologists as to the value of low degree lenses. To assist in solving the problem, will you kindly answer the following questions :

"1. Do you ever prescribe cylinders of less than 0.50 D ? Or spheres of less than 0.75 D ?

"2. If no, have you ever prescribed such lenses, and if so what are the reasons for discontinuing them ?

"3. If yes, in what cases do you prescribe them, and what evidence have you of their value?"

The following is quoted as the result of these investigations :

"Of the one hundred so addressed, seventy-five, located in twenty-six different States, replied. It would seem that these replies must give some indication of the practice of ophthalmologists in general in this country. Most of those addressed were unknown to the writer, except by reputation, and of very few of them was the practice in regard to low degree lenses known. Under these circumstances, it is hardly to be supposed that a great majority of them should have been taken from a minority of oculists. The replies received are surprising, in that they indicate that a very large proportion of oculists employ both weak cylinders and weak spheres ; few only prescribe one but not both, and yet fewer prescribe neither. Thus we find that of the seventy-five, sixty-six replied yes, they do use the 0.25 D. C. and the 0.5 D. S. ; four replied yes, but seldom ; three use the spheres, but not the cylinders, and but two answer, 'No,' and one of those the writer of the editorial in question. Seventy do prescribe the 0.25 D. cylinders, and five do not; and of the five, three prescribe the 0.50 D. spherical.

"In answer to the second question, there are replies from four ; two who say that they do not use the weak cylinders, because they have no evidence of their value, but do not state whether they have ever used them; one who says he has quit, because he is convinced that they are useless, and one who says he rarely uses them now, because he finds that atropia either shows at least 0.50 D. of astigmatism present, or none at all.

"The answers to the third question are fairly uniform, most of the answers being in general terms. That the low degree lenses are prescribed in case where persistent asthenopia, or reflex symptoms, are associated with slight errors of refraction, and the evidence of their value is the disappearance of these symptoms when the proper lenses are adjusted. A number of replies state that in many cases the symptoms remain absent so long as the lenses are worn, and return at once when the lenses are omitted."

In the discussion which followed the reading of this paper, the speakers were nearly all unanimous in expressing a favorable opinion in regard to the benefit to be derived from the prescribing of low degree lenses. The present writer has frequently found great relief accruing to the patient, especially when the tests showed a slight degree of astigmatism, and low degree cylinders were prescribed. The relief which followed, especially from asthenopia, headache, pain in the eye, etc., was more especially marked when the axis of the cylinders varied from the usual vertical and horizontal meridians. The prescribing of lenses should not be done too promiscuously, nor should it be done by any one who is not thoroughly conversant with the physiology and optical principles of the eye, and who does not use all the modern methods for arriving at the errors of refraction. Nothing is to be more deprecated than the way some people have of going into an optical store, picking up various glasses and appropriating that pair which *seems* to afford a better vision, sphericals and cylinders alike, an act which will probably be greatly deplored later on in life. Nor should an ophthalmologist prescribe glasses, especially for a person before the age of fifty, without first having used all the recognized tests for ametropia.

My plan for examination is usually as follows: The vision in both eyes is first ascertained, and then they are both measured with Javal's ophthalmometer for astigmatism. If any astigmatism is found, this is noted for the first test. Next, the refraction is tested by retinoscopy, especially since I place more reliance in it than I do in all other tests, and then by the ophthalmoscope. Finally, the trial lenses are used, according to the findings of the other tests and any variations made that may be found. In addition to all, the muscular equilibrium is tested by means of the red glass, Steven's phorometer, Maddox's rod and double prism. This is the only correct method, if one wishes to obtain satisfactory results.

